# Advancements in DOI-capable TOF-PET modules using a multi-channel high-frequency readout

**DOI:** 10.1186/s40658-026-00870-8

**Published:** 2026-05-04

**Authors:** Giulia Terragni, Elena Tribbia, Joshua W. Cates, Marco Pizzichemi, Johann Marton, Etiennette Auffray

**Affiliations:** 1 European Organization for Nuclear Research, Geneva, Switzerland; 2 Vienna University of Technology, Vienna, Austria; 3 University of Milano-Bicocca, Milan, Italy; 4 Lawrence Berkeley National Laboratory, Berkeley, CA USA

**Keywords:** TOF-PET, High-frequency electronics, Depth-of-interaction (DOI), Light-sharing, Multi-channel readout electronics

## Abstract

**Purpose:**

The achievement of excellent coincidence time resolution has become an essential objective in time-of-flight positron emission tomography (TOF-PET) in order to improve the signal-to-noise ratio and the quality of the reconstructed image. Moreover, to achieve a high level of spatial resolution while maintaining good sensitivity with the use of long scintillators, the impact of the gamma-ray depth of interaction (DOI) must be mitigated.

**Methods:**

To address these challenges, a matrix of sixteen 20 mm-long LYSO:Ce scintillators with depolished lateral surfaces is used, coupled to a light guide that enables light sharing within the matrix. This configuration allows for the determination of the gamma-ray DOI and the correction of its timing bias. For this light-sharing method to work, readout integration in a multi-channel scheme is required. This is achieved using a sixteen-channel low-noise, low-power, high-frequency development circuit board. This high-frequency circuit also provides a solution to enhance the time resolution by enabling a lower leading-edge threshold for the detection of the earliest photons produced with high precision.

**Results:**

The DOI-capable module achieves a DOI resolution of 2.2 ± 0.2 mm full-width-at-half-maximum (FWHM) and a CTR of 133 ± 2 ps FWHM after DOI correction on the central crystals. For comparison, a standard module consisting of sixteen polished crystals and no back light guide achieves a CTR of 130 ± 2 ps FWHM.

**Conclusions:**

Using 20 mm-long LYSO:Ce crystals and this high-frequency electronics, the DOI-capable module delivers excellent timing and energy resolution. In addition to providing time resolution comparable to that of the standard module, it also allows DOI encoding with a resolution of nearly 2 mm FWHM.

## Introduction

High-frequency (HF) front-end electronics [1–3] represent an attractive solution for exploiting the fastest light production mechanisms in scintillating crystals and obtaining excellent performance in time-of-flight positron emission tomography (TOF-PET). Improvement in time resolution will allow for a reduction of the injected radiation dose to the patient, the scan duration, and cost. Alternatively, if these parameters are kept constant, a higher (better) time resolution can lead to an improvement in signal-to-noise ratio, thereby enhancing the quality of the reconstructed image.

HF electronics have demonstrated that time resolution can indeed be significantly improved [2], for they allow a lower leading edge detection threshold, also owing to their low noise, and, therefore, to use the earliest photons produced in the crystals, such as Cherenkov photons emitted in heavy scintillators [4]. HF readout has demonstrated to push the state-of-the-art coincidence time resolution (CTR) to 100 ps using 20 mm-long LYSO:Ce single pixel crystals of 2 $\times$ 2 mm$^2$ cross section [2]. Moreover, as the effect of the depth of interaction of the gamma-ray along the crystal axis (DOI) and the transport of the light in the crystal to the photodetector can cause distortions in the reconstructed images due to parallax errors, the information on the DOI can be retrieved and used to mitigate these effects using different methods: double-side readout methods [5–9], detectors with light sharing DOI encoding [10–12], monolithic scintillators [13], semi-monolithic scintillators [14] and multi-layer detectors [15].

In our approach of extracting the DOI information, we used a matrix of scintillators that are laterally depolished and are coupled, on one side, to an array of silicon photomultipliers (SiPMs) and, on the other, to a light guide that recirculates the light inside the matrix [11]. The DOI coordinate is extracted as the ratio between the light detected by one SiPM, coupled to the scintillator where the interaction occurred, and the total light detected by all the SiPMs. This information can be used to reduce the parallax effect in the reconstructed image and to correct for the bias induced by the DOI on the timing.

In order to benefit from the light-sharing, readout integration in a multi-channel scheme is required. This method, proposed in [11] and investigated in [16, 17], has been shown to provide excellent timing and DOI capabilities. To push the performance even further, in this study, we combine the described detector prototype with a sixteen-channel low-noise low-power HF (LNLPHF) development circuit board and we present the energy, time and DOI resolution achieved.

## Methods

### Detector modules

The modules consist of 4$\times$4 LYSO:Ce scintillators, each with dimensions of 4 $\times$ 4 $\times$ 20 mm$^3$ produced by Crystal Photonics Inc (CPI), coupled via Cargille Meltmount (refractive index n = 1.582) to a 4 × 4 high-performance NUV metal-in-trench (MT) SiPM array [18] from Broadcom, with a total dimension of 16 $\times$ 16 mm$^2$. Each SiPM has a sensitive area of 3.72 $\times$ 3.62 mm$^2$ and a 40 μm SPAD size and is wire bonded, creating non-uniform gaps between the SiPMs.

#### Standard module

The *standard module* makes use of *polished* crystals, each one separated from its neighbors by foils of reflective material (enhanced specular reflector (ESR)). The end of the scintillator block opposite to the photodetectors is also covered by ESR foil, placed in dry contact to act as a mirror.

#### DOI-capable modules

The structure of the *DOI-capable module*, first presented in [11], is very similar to that of the standard module. The differences are that the lateral surfaces of each scintillator are *depolished* and the end of the scintillator block opposite to the photodetectors is coupled to a *light guide* made of glass, using a 150 μm thick layer of optically clear adhesive (OCA), to enable the light recirculation inside the matrix. Finally, the ESR foil is placed in dry contact on the back of the light guide.

**Fig. 1:**
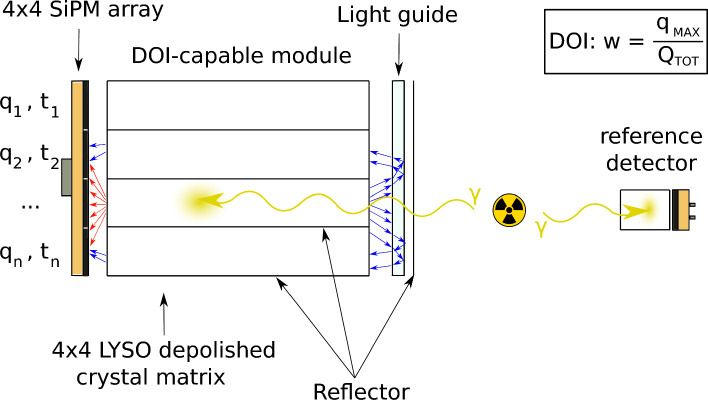
Schematics of DOI-capable modules irradiated frontally

When a gamma ray interacts at a given depth inside the pixel, the light produced propagates towards both the SiPM-side (red arrows in Fig. 1) and the opposite end, i.e. reflector-side (blue arrows in Fig. 1), where, due to the presence of the light guide, it is transported back to the SiPMs by the neighboring crystals.

The depolishing of the lateral sides of the crystals leads to attenuation of the light produced in them. Therefore, the ratio between the amount of light emitted from the two ends of the scintillator allows obtaining the information of the gamma interaction point along the crystal axis through the DOI estimator *w* [19, 20] defined as

1 $$\begin{aligned} w = \frac{q_{max}}{Q}. \end{aligned}$$

$q_{max}$ is the maximum amount of light collected by the SiPM coupled to the scintillator where the interaction occurred, and

2 $$\begin{aligned} Q = \sum _{i=1}^{K} q_{i} \end{aligned}$$

denotes the total amount of light collected by K photodetectors. Moreover, the quantities

3 $$\begin{aligned} u = \frac{1}{Q}\sum _{i=1}^K q_i x_i, \quad v = \frac{1}{Q}\sum _{i=1}^K q_i y_i \end{aligned}$$

are defined using the Anger-logic scheme [16, 21], where $x_i$ and $y_i$ are the coordinates of the centers of the SiPMs, to reconstruct the point of gamma interaction.

### Low-noise low-power high-frequency (LNLPHF) development circuit board

In order to extract the DOI coordinate, the detection of the amount of light hitting each photodetector is required. This can be done by integrating the electrical pulses generated by each photodetector when hit by the scintillation light. At the same time, measuring the time and applying appropriate corrections making use of the DOI information require parallel multichannel readout techniques with fast electronics.

This is made possible by a custom, sixteen-channel development circuit board [22], which is based on the HF signal processing described in [1, 3, 23].

**Fig. 2:**
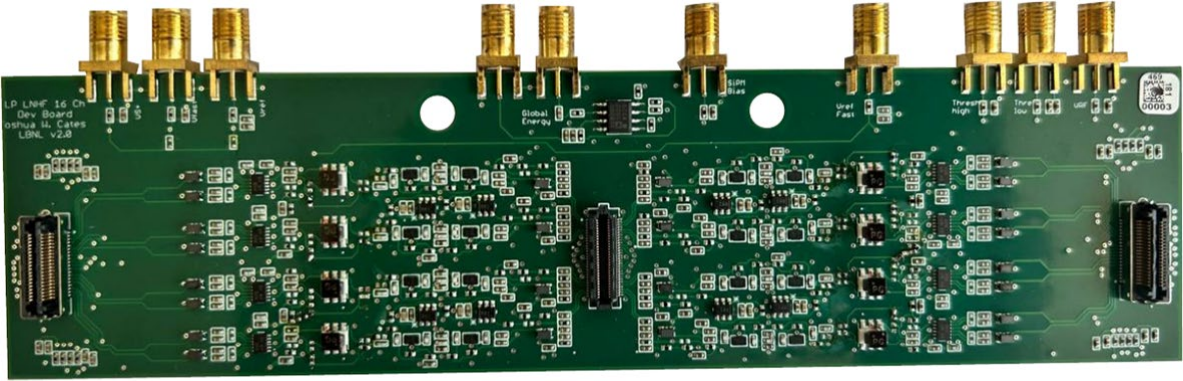
Picture of the sixteen-channel low-noise low-power high-frequency (LNLPHF) development circuit board

This sixteen-channel LNLPHF board, which can host an array of sixteen SiPMs, is shown in Fig. 2. The output of each SiPM is split and processed respectively by a cascade of two radio-frequency amplifiers followed by a fast discriminator to extract a fast-time digital signal and by a low-power operational amplifier to obtain an analog energy signal. A global energy output is also provided by summing the signals from the sixteen SiPMs.

### Data acquisition set-up

The global energy output is used to trigger two CAEN V1742 32-channel digitizers (5 Gs/s, 500 MHz bandwidth) for the digitization and acquisition of the signals. The timing signals are digitized by one of the two digitizers with a precision of a least significant bit (LSB) of 200 ps. The timestamp of the event is derived from the crossing of the rising edge of this digital signal over a fixed threshold.

The energy signals are digitized using the other CAEN V1742 module with a LSB of 400 ps and 1024 bins. The information on the deposited energy is extracted by integrating the signal over all bins.

The boards are received factory calibrated with a time resolution of 4 ps $\sigma$ for a split pulse test, provided that channels of the same DRS4 chip are used. However, the time resolution worsens to 20 ps $\sigma$, when channels of different DRS4 chips are used, due to the time jitter between the chips. The multi-channel board requires digitization of at least 16 channels, thus making use of two or more DRS4 chips. Therefore, the digitizer contributes to the final timing resolution with more than 50 ps full-width-at-half-maximum (FWHM). This contribution is not negligible if CTR values of about 100 ps FWHM are to be achieved. Therefore, a custom calibration as described in [24] is performed. It consists of two calibration steps followed by a synchronization executed during data acquisition. The first calibration addresses the voltage offsets of each cell. To this end, a set of constant reference voltages is injected into every channel of the board and the linear fit of the voltage as a function of the average ADC counts provides the voltage calibration, including offset correction for each cell. The second calibration step addresses the effective time width of each cell. While the factory calibration assumes a fixed cell width of 200 ps, odd and even cells in each channel have different time widths. To measure it, a 50 MHz sinusoidal waveform is injected in each channel and the voltage difference, proportional to the time difference, between adjacent cells is measured. Moreover, a 100 MHz sinusoidal waveform is used to measure the time difference between zero crossings over one or multiple periods and used to correct the time widths of the intermediate cells. Finally, the synchronization of the four DRS4 chips is performed by injecting a 100 MHz sinusoidal waveform into one channel of each group during data acquisition, dedicating 4 channels out of the total 32 to this synchronization procedure. As a result of this dedicated calibration, a time resolution below 6 ps $\sigma$ is achieved both within the same and across different DRS4 chips.

For every valid trigger, the integrated charge and the timestamp are stored for the 16 photodetectors. The value of integrated charge given by each SiPM is corrected for saturation effects due to the finite number of SPADs in the photodetectors. The saturation measurements are performed beforehand using different radioactive sources emitting in the range 30–511 keV.

### Characterization

#### Time and energy performance

**Fig. 3:**
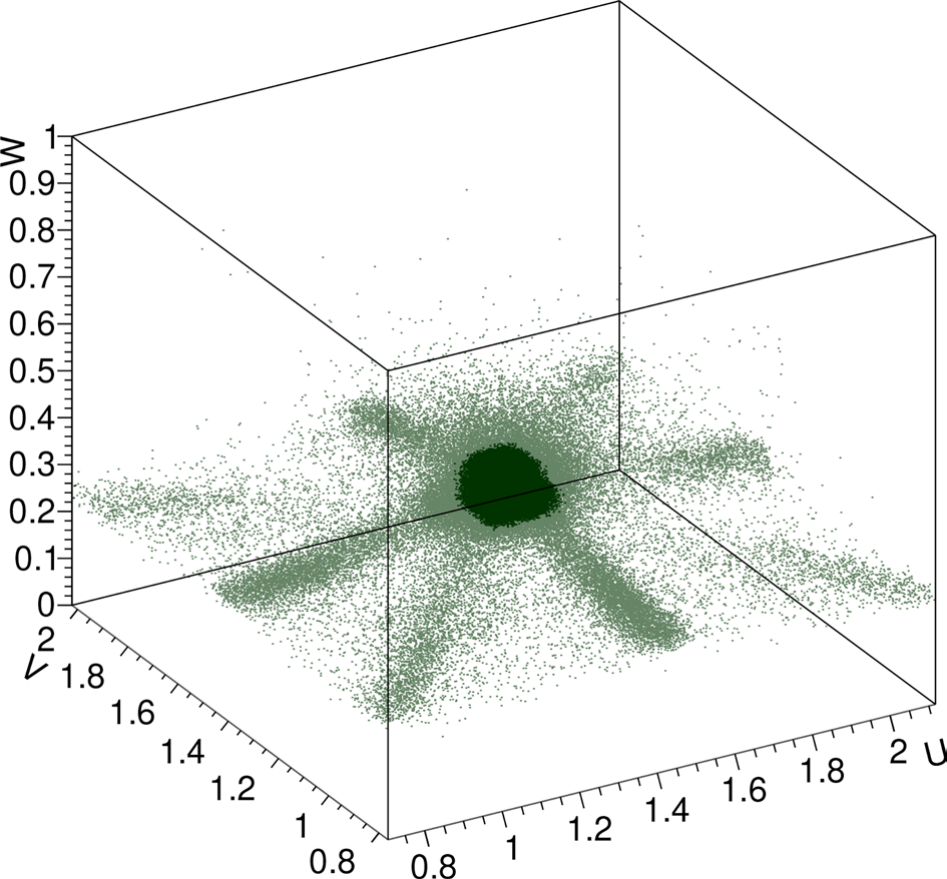
Illustration of one of the sixteen accumulation volumes that can be distinguished in the (*u*, *v*, *w*) coordinate system and selection of the events performed by the clustering algorithm using the DOI-capable module

A PET-like set-up, where the module is frontally irradiated as shown in Fig. 1, is used to run the standard characterization of the module in terms of time and energy resolution. The reference detector is placed in front of the crystal matrix, with the source placed in between such that the entire module is irradiated head-on. A first calibration run is used to record and select the charge spectrum of the reference crystal, removing events outside the 511 keV photopeak. For the remaining coincidence events, the (*u*, *v*, *w*) coordinates are calculated, resulting in sixteen accumulation volumes that can be distinguished. A custom clustering algorithm, as described in [25], is then used to identify and separate the accumulation volumes and events with the full energy deposited in one crystal. Figure 3 illustrates one of the accumulation volumes and the resulting selection of the clustering algorithm. If a standard module were used, the events would be confined within a region close to *w* = 1.0, resulting from the poor light-sharing, whereas in the DOI-capable module, the region is more widely spread out and closer to *w* = 0.0, as shown in Fig. 3.

**Fig. 4:**
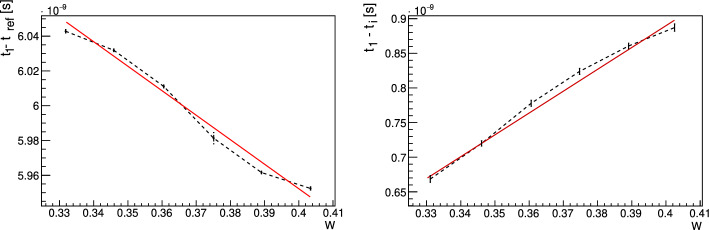
(left) $t_1- t_{ref}$ versus *w* and interpolating function d(w) and (right) $t_1 - t_{i}$ versus *w* and interpolating function g$_i$(w) = [t$_i$-t$_1$](w)

For each volume, the charge spectrum is reconstructed and used to select the region of the photopeak and, at the same time, to evaluate the energy resolution (as FWHM of the Gaussian fit). Once the photopeak events of each crystal are selected, a DOI calibration curve is established for each scintillator. This allows the reconstruction of the physical gamma interaction coordinate from the *w* value, as described in [25] using the gamma attenuation function in the crystal. Finally, a plot of the time difference $t_1-t_{ref}$ versus *w* is produced to derive d(w), the average delay expected as a function of *w* between the detector of interaction D$_{1}$ and the fixed external reference (Fig. 4 left). Moreover, the time delay ($t_1-t_i$) versus *w* is also built for each neighboring detector *i* (Fig. 4 right), to extract g$_i$(w) = [t$_i$-t$_1$](w), i.e. the average delay of the $i$th photodetector with respect to the detector of interaction D$_1$, again as a function of the DOI coordinate *w*.

After the calibration, a similar acquisition run is made and analyzed to assess the timing performance. Only events simultaneously recorded in the photopeak of one of the crystals of the module and of the reference crystal are taken into account. The first delay histogram is constructed without taking advantage of the DOI correction, i.e., taking into account only the difference between the timestamp t$_1$ of the photodetector coupled to the crystal of interaction and the timestamp $t_{ref}$ of the reference crystal. A second histogram is made where one exploits the information from the calibration run in order to correct the timestamp of each neighbouring detector using the functions g$_i$(w). The corrected timestamps are combined to obtain the optimal estimate according to

4 $$\begin{aligned} \hat{t} = \frac{\sum _{i=1}^{16}(1/\sigma ^2) \cdot (t_i - g_i(w))}{\sum _{i=1}^{16}(1/\sigma ^2)} \end{aligned}$$

Finally, the estimator is corrected using the function d(w), which accounts for the delay expected at the measured DOI position w with respect to a fixed reference position w$_0$

5 $$\begin{aligned} \hat{\Theta } = \hat{t} - [d(w)-d(w_0)], \end{aligned}$$

as explained in [16].

The histograms are fitted with an exponentially modified Gaussian distribution to extract the FWHM, which provides the CTR$_{measured}$ of the combined module-reference system. To derive the CTR of the module, the contribution of the reference detector is subtracted as

6 $$\begin{aligned} CTR = \sqrt{2 \cdot CTR_{measured}^2 - CTR_{reference}^2} . \end{aligned}$$

#### DOI performance

**Fig. 5:**
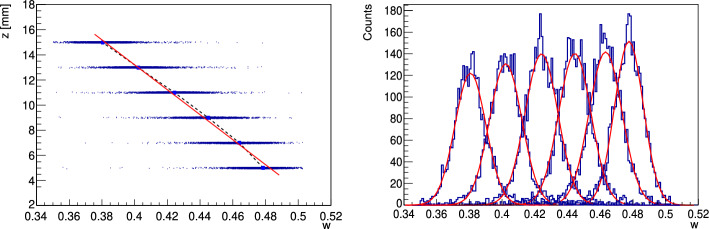
(left) *w* distributions at different known z positions of irradiation along the main axis of the matrix. The experimental relation between *z* and *w* is retrieved. (right) Projections of w distributions

An electronic tagging configuration is used, with the source and the reference detector placed to the side of the module so as to irradiate the crystals laterally [25]. Zaber programmable linear stages are used to precisely align the module with respect to the reference detector. A scan at different DOI positions along the 20 mm long axis of the crystals is made to determine the correlation of the DOI with the *w* coordinate. After applying the clustering algorithm, the distributions of the *w* coordinate corresponding to different DOI positions of the reference crystal are analyzed. The positions of the peak of these distributions show a strong correlation with the z-coordinate and a linear relation between the two variables can be established [25]

7 $$\begin{aligned} z = m \cdot w + q \end{aligned}$$

as illustrated in Fig. 5. Using this relation, which calibrates the w values of each distribution, and summing all events will lead to a distribution that, when fitted with a Gaussian function, yields the crystal DOI resolution in FWHM. Repeating this process for each irradiated crystal, the DOI resolution of the module is then defined as the mean value of all extracted DOI resolutions.

## Results

### Standard module

**Fig. 6:**
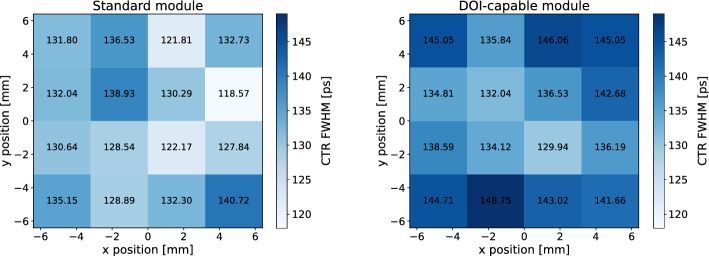
Map of CTR for the 16 crystals of the standard module (left) and DOI-capable module (right)

In order to point out the difference between the standard and DOI-capable module, first, the standard module is measured to test the performance of the described experimental set-up. The module is irradiated head-on, applying a bias voltage of 45 V to the SiPM, corresponding to 13 V of overvoltage, and a 20 mV discriminator threshold that was optimized in previous studies. An average CTR of 131 ± 2 ps FWHM and an energy resolution of 9.5 ± 0.1 $\%$ are obtained over the full matrix. The CTR values for each crystal of the matrix are shown in Fig. 6.

### DOI-capable module

In the ensuing measurement series, the DOI-capable module is measured and first laterally irradiated to examine its DOI resolution following the procedure described in section “DOI performance”. A DOI resolution of 2.2 ± 0.2 mm FWHM is achieved as an average on the central channels (surrounded by 8 neighbouring crystals as would be in a scanner), whereas in the case of all 16 detectors, a resolution of 2.7 ± 0.2 mm FWHM is obtained.

After the DOI measurements with lateral source position, the reference detector and the source are aligned head-on with the module to evaluate its time and energy resolution, as shown in Fig. 1. To demonstrate the effectiveness of the application of the DOI correction, the CTR is evaluated for each channel before (CTR$_{std}$) and after (CTR$_{corr}$) DOI correction, respectively, as described in section “Time and energy performance”. The results are summarized in Table 1 and 2.

**Table 1:** CTR and DOI results from measurements of the DOI-capable module with front and lateral irradiation

Crystals	DOI-capable module			Std. module
	CTR$_{std}$ [ps]	CTR$_{corr}$ [ps]	DOI [mm]	CTR$_{std}$ [ps]
Central	195 ± 2	133 ± 2	2.2 ± 0.2	130 ± 2
All	189 ± 2	140 ± 2	2.7 ± 0.2	131 ± 2

Results from the standard module are shown for comparison. All values are in FWHM

**Table 2:** Energy resolution results using the DOI-capable and standard module with head-on irradiation

Crystals	DOI-capable module	Std. module
	En. Res. [%]	En. Res. [%]
Central	9.6 ± 0.1	9.5 ± 0.1
All	11.3 ± 0.4	9.5 ± 0.1

## Discussion and conclusions

This work presents the measurements made using a standard and a DOI-capable detector module, with the latter being based on the light sharing principle. Both modules are made of 20 mm long LYSO:Ce scintillators and are read out using a state-of-the-art Broadcom MT SiPM array, a sixteen-channel LNLPHF development circuit board and two CAEN V1742 digitizers.

Table 1 and 2 summarize promising results in terms of time, energy, and DOI resolution. If one compares the results obtained on the central channels of the matrix assembly only, it turns out that not only the DOI-capable module achieves a similar CTR as the standard module (133 ± 2 ps FWHM versus 130 ± 2 ps FWHM), but that, in particular, the DOI-capable module has the added benefit of providing DOI information with a resolution as high as 2.2 ± 0.2 mm FWHM. As the CTR is pushed below 100 ps FWHM, the influence of the DOI becomes a dominant contribution to the overall time resolution. In this condition, the DOI-capable module is expected to achieve even better CTR performance than the standard module.

These results should also be seen in the context of previous studies, where other custom-made electronic and commercially available application specific integrated circuits (ASICs) were used. The former comprised the NINO chip, a high-speed amplifier discriminator initially developed for the ALICE experiment [26], and the latter was the PETsys TOFPET2 ASIC [16, 17]. The two systems achieved CTR values of 170 ± 5 ps FWHM and 216 ± 6 ps FWHM, respectively, despite the use of the DOI-module with a more favorable arrangement of shorter (15 mm) LYSO:Ce crystals. By employing longer crystals, this study has demonstrated an even improved coincidence time resolution. Given the excellent CTR achieved, future study will concentrate on the possibility to include the time information to optimize further the DOI parameter w.

Also, compared to the above-mentioned electronics, the LNLPHF readout board minimizes the electronic noise jitter influence on the single photon time resolution, allowing the operation of the detection system at very low thresholds, where even Cherenkov photons can be detected. This capability distinguishes it from the other electronic readouts as demonstrated in studies employing BGO coupled to SiPMs utilizing MT technology [27, 28]. Future tests will investigate matrices composed of different materials, such as BGO and scintillating plastics, as well as heterostructures combining alternating layers of both [5, 29], or GAGG, known to provide both high light output and fast scintillation kinetics [30].

## Data Availability

The datasets generated and analysed during the current study are available from the corresponding author upon reasonable request.
